# Electronic Structure of Oxide Interfaces: A Comparative Analysis of GdTiO_3_/SrTiO_3_ and LaAlO_3_/SrTiO_3_ Interfaces

**DOI:** 10.1038/srep18647

**Published:** 2015-12-22

**Authors:** Hrishit Banerjee, Sumilan Banerjee, Mohit Randeria, Tanusri Saha-Dasgupta

**Affiliations:** 1 Department of Condensed Matter Physics and Material Sciences, S.N. Bose National Centre for Basic Sciences, JD Block, Sector-III, Salt Lake City, Kolkata 700 098, India; 2 Department of Condensed Matter Physics, Weizmann Institute of Science, Israel, 7610001; 3 Department of Physics, Ohio State University, Columbus, OH 43210, United States

## Abstract

Emergent phases in the two-dimensional electron gas (2DEG) formed at the interface between two insulating oxides have attracted great attention in the past decade. We present ab-initio electronic structure calculations for the interface between a Mott insulator GdTiO_3_ (GTO) and a band insulator SrTiO_3_ (STO) and compare our results with those for the widely studied LaAlO_3_/SrTiO_3_ (LAO/STO) interface between two band insulators. Our GTO/STO results are in excellent agreement with experiments, but qualitatively different from LAO/STO. We find an interface carrier density of 0.5 *e*^−^/Ti, independent of GTO thickness in both superlattice and thin film geometries, in contrast to LAO/STO. The superlattice geometry in LAO/STO offers qualitatively the same result as in GTO/STO. On the other hand, for a thin film geometry, the interface carrier density builds up only beyond a threshold thickness of LAO. The positive charge at the vacuum surface that compensates the 2DEG at the interface also exhibits distinct behaviors in the two systems. The compensating positive charge at the exposed surface of GTO charge disproportionates due to correlation effect making the surface insulating as opposed to that in LAO which remains metallic within band theory and presumably becomes insulating due to surface disorder or surface reconstruction.

Following the pioneering work by Ohtomo and Hwang^1^ on LAO/STO, there has been much effort on understanding the interface (IF) between two different insulating ABO_3_ perovskites. The [001] stacking consists of AO and BO_2_ layers, which are charge neutral in one of the oxides like the (SrO)^0^ and (TiO_2_)^0^ layers in STO, but have charge +1/−1 in the other oxides, like (LaO)^+1^ and (AlO_2_)^−1^ in LAO. This creates a polar discontinuity at the interface of the two types of oxides and a build up of electrostatic potential, which can only be averted by a transfer of charge to the interface. The 2DEG that results from this simple *polar catastrophe* picture should lead to an interface carrier density of 0.5 *e*^−^/Ti, corresponding to 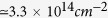, much larger than that achieved in conventional semiconductor hetero-structures.

The *n*-type interface in LAO/STO heterostructures is the most studied of all oxide interfaces^2^,^3^,^4^,^5^. It exhibits gate-tunable superconductivity and in addition shows signatures of local moments and possible ferromagnetism^6^,^7^,^8^,^9^ coexisting with the superconductivity. The density of itinerant carriers at the interface, however, is consistently found to be an order of magnitude smaller^10^,^11^,^12^ than 0.5 *e*^−^/Ti, the value expected from the polar catastrophe model. In addition, the interfaces are insulating, rather than being metallic, below a certain critical thickness of LAO layers^12^.

A more recent development is the study of the *n*-type interface between the Mott insulator GTO and the band insulator STO grown by molecular beam epitaxy^13^,^14^,^15^. Note, GTO with Ti ions in 3+ state, is in *d*^1^ state as opposed to Al states of LAO which are empty. This hints towards the crucial role of Ti *d* electrons and potential correlation effects in making a difference between GTO/STO and LAO/STO. Remarkably, the GTO/STO samples give rise to 2DEGs with carrier densities of 0.5 *e*^−^/Ti, exactly as expected from the ideal polar catastrophe scenario. Furthermore, the GTO/STO interface is found to be conducting irrespective of layer thickness of GTO, so that there is no thickness threshold for metallic behavior. In both these respects GTO/STO seems to be qualitatively different from LAO/STO. The 2DEG at the GTO/STO interface also shows many other interesting properties^13^,^14^,^15^, including possible transport signatures for magnetism^16^ and strong correlations^17^.

Motivated by these developments, we present here a detailed electronic structure study of the GTO/STO interface and contrast our results with those obtained for LAO/STO. While the LAO/STO interface has been thoroughly studied by electronic structure calculations^18^,^19^,^20^,^21^, much less is known about GTO/STO. The specific problem of single SrO layer in a GTO matrix in the superlattice geometry has been studied^22^,^23^,^24^ by a variety of techniques, first-principles, model Hamiltonian as well as combined density functional theory (DFT) and dynamical mean field theory. There have been some suggestions^25^ about the origin of the differences between the LAO/STO and GTO/STO systems, but to the best of our knowledge, no first principles electronic structure study exists which compares the LAO/STO and GTO/STO interfaces on same footing in different heterostructure geometries. Gaining insight into GTO/STO and into the differences and similarities with LAO/STO is very important for the advancement in the field of oxide interfaces. We provide a simple, intuitive understanding of our detailed DFT results at the end of the paper, summarized in terms of a schematic diagram and the associated discussion.

We conclude the introduction by summarizing our main results for *n*-type interfaces in GTO/STO and LAO/STO in two different geometries: (a) a superlattice and (b) a thin film on an STO substrate with vacuum on top. (1) Both GTO/STO and LAO/STO show essentially similar behavior in the superlattice geometry, despite differences in details of the orbital character of the carriers due to different structural distortions. The key result is that both GTO/STO and LAO/STO superlattices have an interfacial charge density of 0.5 *e*^−^/Ti, and there is no critical thickness of GTO or LAO for conductivity in the superlattice geometry. (2) The behavior of the two systems is very different in the thin film-substrate geometry. We find that GTO/STO conducts even for GTO thickness of 1 unit cell, the minimum thickness possible, while LAO/STO conducts only beyond a critical thickness of 5 unit cells of LAO. (3) In the thin film-substrate geometry, GTO/STO has an interface carrier density of 0.5 *e*^−^/Ti independent of GTO thickness. In contrast, the carrier density in LAO/STO is about a factor of four smaller just beyond the threshold for conductivity, and rises slowly with increasing LAO thickness. (4) We find that, in the thin film geometry, the surface layers facing the vacuum have very different electronic structures in LAO/STO and GTO/STO. The holes at Ti sites on the top GTO layer charge disproportionate making the surface insulating, while the holes on top LAO layer are metallic within band theory.

## Results

### Heterostructure Geometries

**Figure 1:**
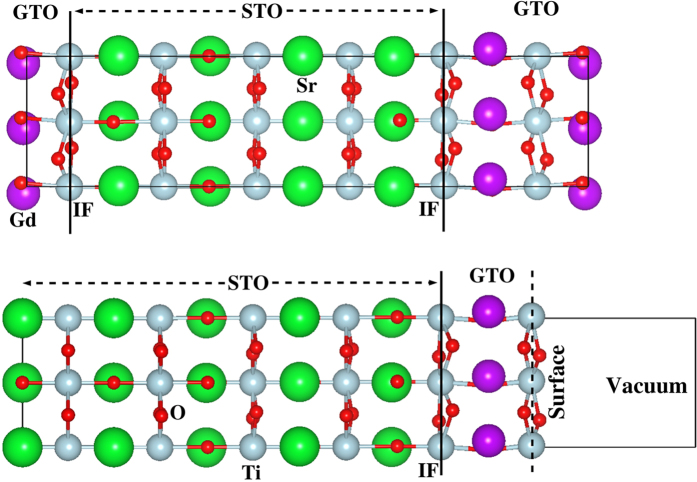
The two geometries used in the present study: superlattice (upper panel) and thin film-substrate (lower panel). We show the representative cases of (GTO)_1.5_/(STO)_4.5_ superlattice and (GTO)_1_/(STO)_5_ thin film-substrate geometry. The various atoms are: Gd (purple), Ti (grey), O (red), and Sr (green). The interfaces (IFs) formed between GdO from GTO and TiO_2_ from STO are marked.

A study of both experimental and theoretical literature^1^,^2^,^3^,^13^,^14^,^15^,^26^,^27^,^28^ shows that the oxide interfaces have been investigated in two different geometries, (i) superlattice geometry with periodic repetition of alternating layers of STO and, LAO or GTO, and (ii) thin film of LAO or GTO grown on a STO(001) substrate. Most experimental studies on LAO/STO are carried out in a thin film-substrate geometry, while both geometries have been investigated in GTO/STO experiments. Since we would like to have a comparative study of the two systems with an aim to arrive at a common understanding, in the present study we consider both the geometries, as shown in Fig. 1. Following the experimental literature on GTO/STO^13^,^14^,^15^, we consider only *n*-type IFs formed between GdO layer from GTO and TiO_2_ layer from STO in GTO/STO, and between LaO and TiO_2_ in LAO/STO. In the superlattice geometry, we consider two symmetric *n*-type interfaces in the cell which result in non-stoichiometric supercells with an additional TiO_2_ layer in STO and an additional GdO layer in GTO (LaO layer in LAO). This results in superlattices with the formula (LAO)_*p*.5_/(STO)_*q*.5_ or (GTO)_*p*.5_/(STO)_*q*.5_. Calculations are carried out for *p* = 1, 2, 3, 4 and *q* = 4 and 9.

The thin film-substrate geometry, shown in the bottom panel of Fig. 1, creates a single *n*-type interface and a surface of TiO_2_ (in case GTO/STO) or AlO_2_ (in case of LAO/STO) facing the vacuum. Within the periodic set-up of DFT calculations, this amounts to having periodic copies of STO/(LAO or GTO) heterostructures separated by a vacuum layer. The general formula of the thin film-substrate systems is (LAO)_*p*_/(STO)_*q*_ or (GTO)_*p*_/(STO)_*q*_. Calculations are carried out for *p* = 1, 2, 3, 4, 5, 6 and *q* = 9.

The in-plane dimension of the simulation cell is expanded by 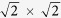 creating two Ti or Al atoms in the BO_2_ layers of the unit cell to take into account the GdFeO_3_-type orthorhombic distortion characterized by tilt and rotation of the TiO_6_/AlO_6_ octahedra. This becomes specially important for the GTO/STO system, as we will see in the following.

### Structure

**Figure 2:**
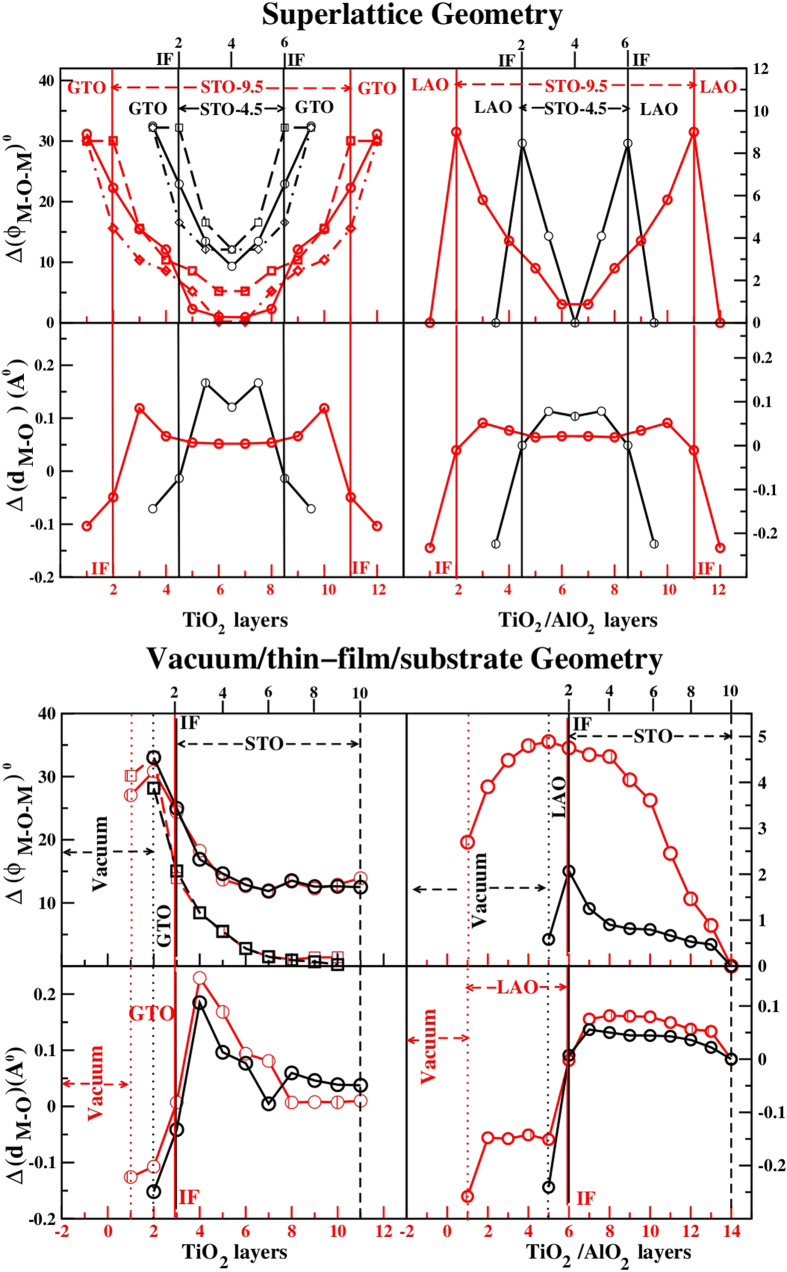
The deviation of M-O-M angles from 180°, Δ (*ϕ*_*M*−*O*−*M*_) and the difference of M-O lengths in the out-of-plane and in-plane directions, Δ (d_*M*−*O*_). On the right we show results for LAO/STO and on the left for GTO/STO. For superlattices (upper panels), we show results for (LAO or GTO)_4.5_/(STO)_1.5_ (black symbols) and (LAO or GTO)_9.5_/(STO)_1.5_ (red symbols). For the thin film-substrate geometry (lower panels), results are shown for (GTO)_1_/(STO)_9_ (black symbols), (GTO)_2_/(STO)_9_ (red symbols), and (LAO)_1_/(STO)_9_ (black symbols), (LAO)_5_/(STO)_9_ (red symbols). The x-axis labels for the red curves are marked in red at the bottom of the figures, while those for the black curves are marked in black at the top. For GTO/STO, we show the rotations (circles) and the asymmetric tilts in +*c* (squares) and -*c* (diamonds) directions; see text. For LAO/STO only the rotation angles are shown as circles, the tilt angles being zero.

We first start with discussion of the structural properties of the studied heterostructures. As already mentioned, the presence of GdFeO_3_-type orthorhombic distortion is an important structural aspect of GTO. This distortion in bulk GTO makes the structural properties of the optimized GTO/STO systems rather different compared to LAO/STO. Structural distortions observed include the tilt and rotation of the metal (M) - oxygen(O) octahedra as well as the compression or elongation of the individual MO_6_ octahedra. Figure 2 shows the plots of the deviation of M-O-M bond angle from 180°, as well as the difference between out-of-plane and in-plane M-O bond lengths. The former quantifies the tilt/rotation of MO_6_ octahedra, while the latter quantifies the compression (for negative sign) or elongation (for positive sign) of MO_6_ octahedra. The top panels of the figure show the result for the superlattice geometry while the bottom panels are for the thin film-substrate geometry. The qualitative behavior is similar between the two geometries.

For the GTO/STO systems, the deviation of the Ti-O-Ti bond angle from 180° is as high as 30° or so in the GTO side. This decreases systematically and reaches a value of about 5°–10° within the interior of STO block. For thin film-substrate geometry the tilt/rotation attains a constant value within the interior of STO, which is found to be substantial for rotation. The TiO_2_ layer at the IF faces the SrO layer on one side and GdO on the other. This makes the tilt angle in the 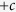 and 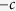 directions with respect to the TiO_6_ octahedra to be different (*c*-axis is the [001] direction that is perpendicular to the IF). This effect is found to percolate to other TiO_2_ layers as well, specially in the case of GTO/STO. At the interfaces, the highly asymmetric out-of-plane tilt angles vary between about 15° and about 25–30°. The in-plane and out-of-plane Ti-O bondlengths become unequal in GTO layers, with maximum difference of 0.1–0.2 *Å*, indicating distortion of the TiO_6_ octahedra. This distortion becomes smaller at IF and inside the STO block it attains a value of ≈0.1 *Å* or smaller. The MO_6_ octahedra are compressed in GTO layers, and are elongated in STO block. For the thin film-substrate geometry, the distortion of TiO_6_ attains more or less a small constant value inside the interior of STO block.

In comparison, in LAO/STO, the deviation of M-O-M bond angle from 180° occurs only for rotation, which is much smaller in magnitude compared to GTO/STO. The rotation angles are only significant at the IFs or close to them with values of about 5–9°. The tilt angles are found to be zero. Like in GTO/STO, the metal-oxygen octahedra are compressed in LAO side and elongated in STO side. The structural differences between GTO/STO and LAO/STO, specially in terms of tilt and rotation of MO_6_ octahedra, has important bearing on the orbital character of the conducting electrons at different layers, as will be elaborated in the following.

### Electronic Structure

**Figure 3:**
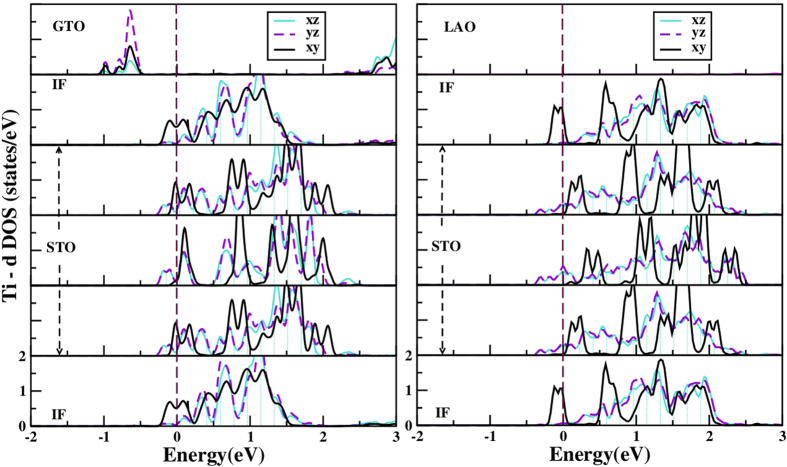
The density of states projected to *xy*, *xz*, *yz* orbitals of Ti, in different TiO_2_ layers of (LAO)_1.5_/(STO)_4.5_ (right panels) and (GTO)_1.5_/(STO)_4.5_ (left panels) superlattices. The top panel corresponds to TiO_2_ in GTO on the left, and to AlO_2_ layer in LAO on the right (for which there are no states in the energy range shown). The subsequent panels from top to bottom (both on the left and right) refer to TiO_2_ at 1st IF, 1^*st*^, 2^*nd*^ and 3^*rd*^ TiO_2_ layers in STO block, and TiO_2_ at the 2^*nd*^ IF in the cell. The cell is symmetric about the 2nd TiO_2_ layer in STO. The zero of the energy is the Fermi energy.

We analyze the electronic structure of the optimized GTO/STO and LAO/STO heterostructures in both superlattice and thin film-substrate geometry in terms of density of states, charge and orbital populations. Figure 3 shows the layer-wise density of states (DOS) projected to Ti 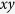, 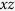 and 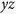 states in (GTO)_1.5_/(STO)_4.5_ and (LAO)_1.5_/(STO)_4.5_ superlattices. Qualitatively similar results are obtained for (GTO)_1.5_ or (LAO)_1.5_/(STO)_9.5_ superlattices, proving that the physics in the superlattice geometry is independent of the STO thickness. First, we find both GTO/STO and LAO/STO superlattices are metallic with non-zero density of states at the Fermi level (E_*F*_). Since the layer thickness of GTO or LAO of 1.5 layers is the minimum possible within the superlattice geometry, we conclude that for both LAO/STO and GTO/STO superlattices, there is no minimum thickness for conductivity. As expected, calculations with larger thickness of GTO and LAO (checked with thicknesses of 2.5, 3.5 and 4.5 unit cells), are also found to be metallic.

We find that in both superlattices, the conducting electronic charge is not strictly confined to the IF, and spreads out into several layers of the STO block. This is in agreement with experimental findings^29^, and previous theoretical studies^18^ of LAO/STO. We note, however, that the nature of the conduction electrons is different in the two superlattices, as seen from the DOS in Fig. 3. We find that, while the carriers at the IF are predominantly of 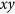 character for both GTO/STO and LAO/STO, the situation is different in two systems in the TiO_2_ layers in STO, specially those adjacent to IF. The orbital characters of the carriers in these layers are mixed in case of GTO/STO, and 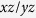 type in case of LAO/STO. This is further corroborated by the plots of conduction electron charge densities, as shown in Fig.S1 of the supplementary information (SI) for (LAO)_1.5_/(STO)_4.5_ and (GTO)_1.5_/(STO)_4.5_. This difference stems from the structural differences between GTO/STO and LAO/STO.

To obtain the layer-wise contribution to the conduction electron, we integrate the layer projected DOS from 0.5 eV below E_*F*_ to E_*F*_. For GTO/STO system this corresponds to integrating from the upper edge of the lower Hubbard band of Ti *d* in insulating GTO layer to E_*F*_. The electron from Ti^3+^ ion in GTO layer in its 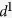 charge state occupies the localized lower Hubbard band and does not contribute to conduction. Table 1 shows the layer-wise contribution to the conduction electron for 1.5/4.5 as well as 1.5/9.5 superlattices. We find the total conduction charge in the LAO/STO as well as in GTO/STO superlattices to be 1 *e*^*−*^ irrespective of the thickness of STO layers. This is fully consistent with the presence of two symmetric interfaces each with a carrier density of 0.5 *e*^−^/Ti. Interestingly we find Ti *d* states at the IF of GTO and STO are spin-polarized, with Ti *d* states within STO layers adjacent to IF inheriting this spin-polarization, as shown in Fig. S2 in SI. Moving further away from the IF, the spin-polarization decreases and finally vanishes deep inside the STO block. The calculated magnetic moments at Ti sites are found to be ≈0.15 *μ*_*B*_, ≈0.12 *μ*_*B*_ in the STO layer next to IF, ≈0.02 *μ*_*B*_ in the following layer, and vanishingly small in other layers. The Ti moments are found to be aligned in a ferromagnetic arrangement, consistent with experimental results^30^.

**Figure 4:**
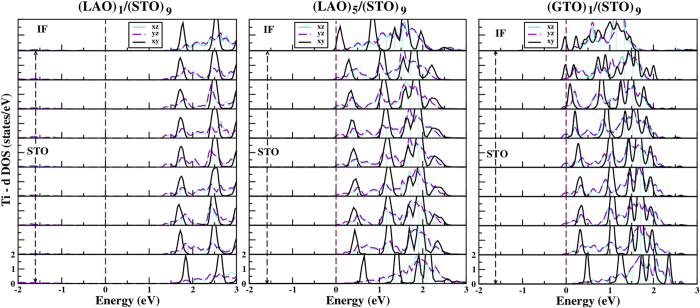
The density of states projected to *xy*, *xz*, *yz* orbitals of Ti, in different TiO_2_ layers of LAO/STO and GTO/STO in thin film-substrate geometry. From top to bottom, different panels refer to TiO_2_ at IF, TiO_2_ layers belonging to STO block. The zero of the energy is set at E_*F*_.

A similar analysis in the thin film-substrate geometry shows dramatically different behavior. The left and middle panels in Fig. 4 show the DOS in different TiO_2_ layers of LAO/STO in thin film-substrate geometry with two different thicknesses of LAO layers, 1 and 5 unit cells, respectively. We find that the IF in 1 unit cell of LAO on STO is insulating with a large gap between the valence and conduction states, and no states at E_*F*_. On the other hand, 5 unit cell of LAO on STO is barely metallic, setting a critical LAO thickness of 5 unit cell for the conductivity. This behavior is significantly different from that of LAO/STO in superlattice geometry for which IFs are found to be conducting for any thickness of LAO. This difference in conduction properties of LAO/STO, depending on the system geometry has been pointed out previously in literature^20^,^21^.

A markedly different picture is obtained for GTO/STO system. The right most panel of Fig. 4, shows the plot of density of states of GTO/STO in thin film-substrate geometry with 1 unit cell thickness of GTO. We find the solution to be metallic even in the limit of 1 unit cell thickness of GTO. This is in sharp contrast to LAO/STO case, but in excellent agreement with experimental reports on GTO/STO^13^. The calculation of total conduction charge by integrating the layer wise density of states from −0.5 eV below E_*F*_ to E_*F*_ gives a charge of 0.5 *e*^−^ (see Table 1) for GTO/STO in thin film-substrate geometry with 1 unit cell thickness of GTO. This is in complete accordance with a single *n*-type interface in the unit cell, and the expectation from polar catastrophe model. In contrast, the total conduction charge for the LAO/STO in thin film-substrate geometry with 5 unit cell thickness of LAO, which is at the critical thickness of metallicity, is found to be about 0.14 *e*^−^/Ti, almost a factor of 4 smaller than 0.5 *e*^−^/Ti expected from the ‘ideal’ polar catastrophe scenario. Increasing the LAO thickness beyond 5 u.c., the carrier concentration is found to slowly increase, for example for (LAO)_6_/(STO)_9_ the conduction charge is found to be 0.18 *e*^−^. This is expected to reach the asymptotic value of 0.5 *e*^−^ for very large thickness of LAO, as discussed below. Similar to the observation in superlattice geometry, we find the Ti *d* states to be spin-polarized at the IF of GTO and STO, with spin-polarization of Ti *d* states percolating down in the STO block (cf Fig. S2 in SI). The estimates of the magnetic moments are found to be similar to that in case superlattice geometry, with parallel alignment of spins.

The analysis of orbital population of the conduction electron shows the carriers at the IF are predominantly of 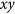 character, while that within the STO block to be predominantly of 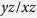 character in LAO/STO with LAO thickness beyond the critical thickness of conductivity. For GTO/STO, the IF is of significant 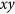 character, the subsequent layers being of mixed character which converts to predominant 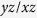 character in the interior of STO block. We thus find that the orbital characters in LAO/STO and GTO/STO are quite similar in the superlattice and thin film-substrate geometries, as might be expected given the similarities in the structural distortions in the two geometries.

**Figure 5:**
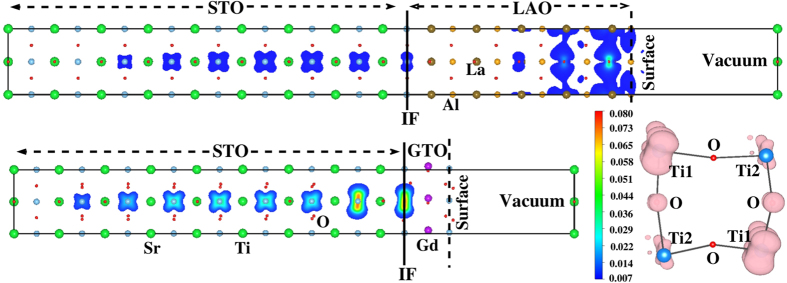
Conduction electron charge density for (LAO)_5_/(STO)_9_ (upper panel) and (GTO)_1_/(STO)_9_ (lower panel) in thin film-substrate geometry, integrated over an energy window from 0.1 eV below E_*F*_ to E_*F*_. The color of different contours corresponds to the values shown in the scale bar. The topmost AlO_2_ surface layer in LAO is metallic, while the topmost TiO_2_ layer in GTO is insulating. Inset: We also show the charge disproportionation with two inequivalent Ti sites on the top layer of GTO which drives the surface insulating. The charge density in this plot is obtained from integration over the lower Hubbard band in GTO; see text for details.

Another pertinent issue in the context of thin film-substrate geometry is the fate of the surface layer facing the vacuum, which is AlO_2_ in LAO, or TiO_2_ in GTO. By simple charge balance, the uppermost surface layer should be hole doped to compensate for the electrons at the n-type interface. For instance, the surface layer in GTO should have Ti in a 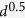 state, rather than the 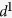 state in bulk GTO. This simple picture, of course, does not take into account the disordering effects like oxygen vacancies and cation disorder, or the effect of surface reconstruction, which are reported to be important in the context of LAO/STO^31^,^32^. Interestingly, our DFT calculation which allows for possible structural reconstruction only within the scope of 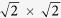 cell, shows the topmost AlO_2_ surface in LAO to be metallic, while the topmost TiO_2_ surface in GTO to be insulating. This is seen in the plot of charge density contributed by a narrow energy window around E_*F*_, for (LAO)_5_/(STO)_9_ and for (GTO)_1_/(STO)_9_ (cf Fig. 5). While the surface reconstruction in reality can be complex, which undoubtedly needs further exploration both from experimental and theoretical side, the stabilization of the insulating solution at the topmost TiO_2_ surface layer of GTO doped with 0.5 hole is interesting. We find this to be triggered by charge disproportionation between two Ti atoms at the top layer. With the choice of *U* = 7 eV on Ti atoms (see Methods for further discussion), this charge disproportionation becomes complete leading to insulating solution with nominal charge of 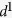 on one Ti atom and 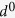 on the other, maintaining an average charge of 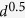 per Ti at the top layer. This is evident from the charge density plot focused on the energy window around the occupied lower Hubbard band (LHB) of Ti *d* states (see inset in Fig. 5), which shows significantly large charge on one set of Ti atoms and a significantly smaller on the other. We find this disproportionation to be triggered by the strong correlation effect which together with small differences in local environment of two Ti atoms, make the charges on two Ti’s significantly different. The associated magnetic moments at two charge disproportionated Ti sites are found to be ≈1 *μ*_*B*_ and ≈0 *μ*_*B*_, with spins of magnetic Ti ions feromagnetically aligned.

**Figure 6:**
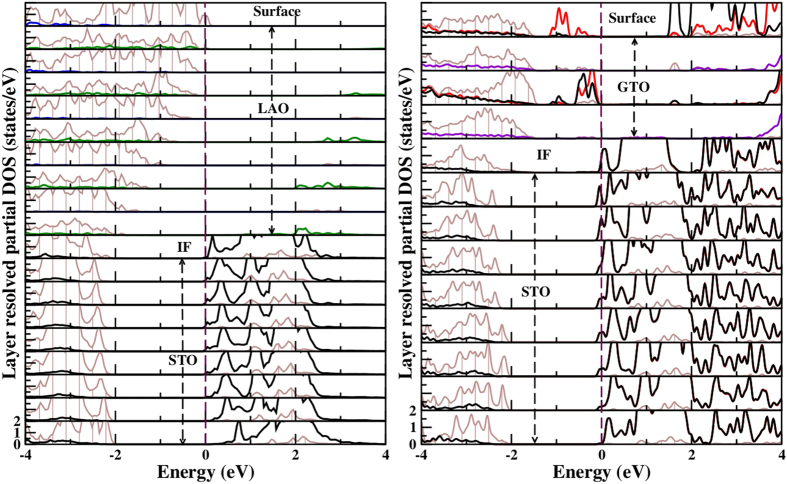
The layer decomposed DFT density of states for (LAO)_5_/(STO)_9_ (left panel) and (GTO)_2_/(STO)_9_ (right panel) in thin film-substrate geometry, projected onto O *p* (brown line), Al *p* (green line), Ti *d* (black/red line) and Gd *d* (magenta) and La *d* (blue line) states. For GTO/STO, the projection to two charge disproportionated Ti atoms are shown as black and red lines, respectively, while for LAO/STO projection to Ti *d* is shown as black line. E_*F*_ is set at zero. From top to bottom, various panels show the surface layer, the LAO or GTO block, the IF and the STO block.

We show the DFT layer-resolved density of states, projected onto O *p*, Ti *d* and Gd *d* states, for (GTO)_2_/(STO)_9_ in the thin film-substrate geometry in the right panel of Fig. 6. To clearly show the charge disproportionation in this plot, we plot the DOS contributions for the two inequivalent Ti atoms at the surface using two different colors in Fig. 6. The disproportionation weakens as we move away from the vacuum surface towards the interface and is absent at the IF. We note that the charge disproportionation found at the surface does not lead to an additional translational symmetry breaking, associated with the opening of the charge gap, given that a 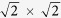 structural distortion is already present in bulk GTO.

### Electronic Reconstruction

Our DFT results described above lead to the following conclusions.

1. Both GTO/STO and LAO/STO show essentially similar behavior in the superlattice geometry, despite differences in details of the orbital character of the carriers due to differences in the structural distortions. The central result is that both GTO/STO and LAO/STO superlattices have the full interfacial charge density of 0.5 *e*^−^/Ti and there is no critical thickness of GTO or LAO for conductivity.
2. The behavior of the two systems differs qualitatively in the thin film-substrate geometry. We find that GTO/STO conducts with an interfacial carrier density of 0.5 *e*^−^/Ti, independent of GTO thickness. In contrast, LAO/STO conducts only beyond a critical thickness of 5 unit cells of LAO, and the interfacial carrier density is still almost a factor of four less than 0.5 *e*^−^/Ti just beyond threshold.

We now address the question: What are the driving mechanisms behind the similarity and differences between the two geometries in the two systems? In the superlattice geometry, we need an extra GdO or LaO layer with a charge of +1, as emphasized by Chen *et al.*^21^. This is then directly responsible for doping the two (symmetric) *n*-type interfaces with 0.5 *e*^−^/Ti each. In effect, there is no polar catastrophe (potential divergence) that is averted in this geometry; rather one simply obtains a fixed interface carrier concentration, independent of the thickness of GTO or LAO, as demanded by charge neutrality.

However, in the film-substrate geometry with a single interface, we find that band alignment, band bending and electronic reconstruction play a crucial role. As a result, there are striking differences between LAO/STO and GTO/STO. To understand these differences, it is useful to look at the DFT results for the density of states (DOS) in the thin film geometry.

**Figure 7:**
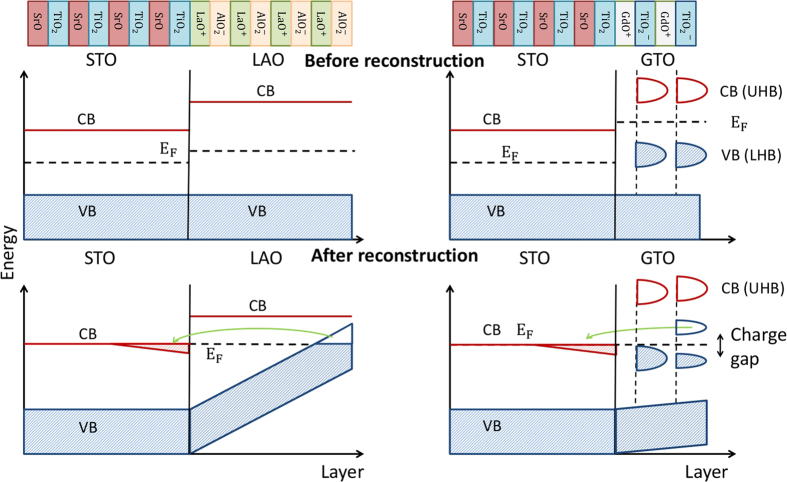
Schematic plot of the band offset (top panels) and electronic reconstruction (bottom panels) that summarizes some of the important DFT results described in the text. The left panels show results for LAO/STO, while GTO/STO results are shown on the right. Prior to electronic reconstruction in LAO/STO, the top of the valence band (VB) in LAO is widely separated in energy from the bottom of the conduction band (CB) in STO. Thus electronic reconstruction requires a large band bending, which is responsible for the threshold in LAO thickness for the polar catastrophe to set in. In contrast, the VB in GTO is the Ti-d lower Hubbard band, which is already close in energy to the CB in STO prior to reconstruction. Thus only a modest band-bending is required for a conduction electron density of 0.5e per interface Ti. We also see in the reconstructed GTO/STO electronic structure the charge gap due to the correlation-induced charge disproportionation in the top TiO_2_ layer next to vacuum.

The left panel in Fig. 6 shows the density of states plotted over a wide energy window, projected onto O 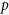, Al 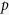, Ti 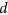 and La 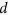 states for (LAO)_5_/(STO)_9_ in the thin film-substrate geometry. This provides the picture after electronic reconstruction as given by DFT. We see that the upper edge of the oxygen valence band (VB) in LAO bends progressively towards E_*F*_, moving from the interface to the surface. We see that the surface AlO_2_ layer next to vacuum is hole doped into a metallic state with electrons transferred from the surface to the conduction band (CB) of Ti *d*-states at the IF. This can be seen schematically in the lower left panel of Fig. 7.

The band alignment before electronic reconstruction can be derived from the bulk band structure of LAO and STO shown schematically in the upper left panel in Fig. 7. The experimentally measured bulk band gap of STO is 3.3 eV, while that of LAO is 5.6 eV. In LAO/STO, the valence band offset is small, and the VB maxima of LAO and STO, which is the upper edge of filled O 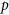 bands, are almost aligned. For the electronic reconstruction, therefore a large band bending with VB maxima of LAO at the surface aligning with conduction band minima of STO is needed, as observed in DFT results presented in Fig. 6 and schematically in left lower panel of Fig. 7.

The necessary band bending in LAO/STO is estimated to be about the same as band gap 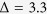 eV of STO. One can make a simple estimate^20^,^33^ of the critical thickness 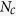 of LAO layers for charge transfer from the surface to the interface as follows. The potential difference between the surface and interface is 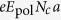, due to the polar field 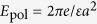 (where *ε* is the dielectric constant of LAO arising from both electronic and ionic screening) should be equated to the gap Δ. Using the bulk dielectric constant of LAO, 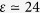, and the in-plane lattice constant of 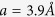, the critical thickness could be estimated to be 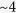 layers, consistent with experiment and the DFT result. For thickness 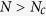, when an amount of charge 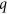 is transferred to the interface the potential difference 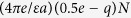 stays pinned at Δ. This leads to an estimate of 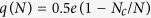, that approaches the asymptotic value of 0.5 *e* for 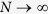. For 5 LAO layers, one obtains 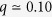, roughly consistent with the DFT result of Table 1.

The electronic reconstruction scenario is quite different for GTO/STO. Bulk GTO is a Mott insulator and the relevant valence band is the Ti *d* lower Hubbard band (LHB), which lies far above the O *p* derived VB of the STO, as shown schematically in the right upper panel in Fig. 7. The Ti *d* LHB lies only 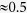 eV below the conduction band of STO. Electrostatic considerations, similar to the ones discussed above for LAO/STO, with the bulk dielectric constant of 30 for GTO imply that a minimum thickness of 1 u.c. of GTO is sufficient to allow for required band bending and charge transfer. This would suggest the charge is transferred to the interface independent of the thickness of the GTO layers, consistent with our DFT results and also with the experimental observations^13^,^14^. Apart from the usual upward bending of the GTO VB approaching the surface, the Ti *d* LHB in GTO also bends upward near the interface to connect with the Ti *d* derived conduction band in the STO side, as seen in Fig. 6 (right panel).

Finally, we stress again the fate of the holes on the top surface layer next to vacuum, that must exist to counterbalance the electrons at the interface. Unlike LAO, which is a band insulator, GTO is a correlation driven Mott insulator in the bulk. As described above, we find a rather strong correlation-induced charge disproportionation on the topmost TiO_2_ layer of GTO at the surface. We thus find the LHB of Ti *d* states at the top layer of GTO, which would naively have been partially filled (average filling of *d*^0.5^) and metallic, splits into occupied and unoccupied bands due to opening of a charge gap due to the charge disproportionation. This is clearly seen in the DFT result of Fig. 6 (right panel) and shown schematically in the lower right panel of Fig. 7. Our theoretical observation of charge disproportionation in top TiO_2_ layer in GTO/STO in thin film-substrate geometry should be explored experimentally.

## Discussion and Outlook

In this work we have carried out a detailed first-principle DFT study of LAO/STO and GTO/STO heterostructures, focusing on their essential similarities and differences in two experimentally well-studied geometries, namely the superlattice and thin film-substrate geometry. While the two systems behave quite similarly in the superlattice set up, e.g. in terms of the total 0.5 *e*^−^/Ti transfer of charges to the interface, very different pictures emerge in case of thin film-substrate geometry due to the differences in electronic reconstructions in the two systems, even though, in both the cases, the reconstructions are driven by the same underlying electrostatic mechanism, namely the need to avert the ‘polar catastrophe’. We find a full 0.5 *e*^−^/Ti conducting charge at the interface even for 1 u.c. thick GTO on STO substrate, consistent with experiments^13^,^14^. On the other hand, in case of LAO on STO, the transferred charge only increase gradually with thickness from a small value of ~0.14 e^−^/Ti above a critical thickness of about 4.

Additionally, in the thin film-substrate case, we find the fate of the surface layers, which host the neutralizing charges for the interface carriers, to be quite distinct. The electronic states derived from O p orbitals at the surface LAO layer turn out to be metallic within DFT but are experimentally^31^ found to be localized, possibly due to disorder or surface imperfections like oxygen vacancies^32^. On the other hand, the Ti *d* states at the top most layer of GTO/STO correspond to a doped Mott insulating layer of GTO and stays insulating by opening a charge gap via an interesting correlation driven charge disproportionation. Such a correlation induced phenomenon could be a robust feature, at least within a short length scale, even in the presence of surface disorder and, in principle, could be probed experimentally. This also pertains to the experimental verification of the polar catastrophe mechanism by detecting the counter charges at the surface of the heterostructure. The presence of conducting charges at the interface by itself does not ambiguously establish the polar catastrophe mechanism for the polar oxide interfaces as there could be other possible sources of interfacial charge carriers, e.g. oxygen vacancies^1^.

A few interesting future directions for theoretical study would be to investigate in detail the sub-band structures, that is relevant for quantum oscillation measurements^15^, and spin-orbit coupling in the GTO/STO interface. Our structural analysis indicates substantially larger polar distortions of the Ti-O-Ti bonds at the GTO/STO interface compared to that in LAO/STO. This could potentially lead to much larger Rashba spin-orbit coupling for GTO/STO heterostructure. The magnetic property of the interface 2DEG in GTO/STO should also be investigated by DFT. GTO/STO shows signature of ferromagnetism that could be an intrinsic correlation driven phenomenon, independent of the proximity to magnetic GTO layer^16^.

## Methods

Our first-principles calculations are based on plane wave basis as implemented in the Vienna Ab-initio Simulation Package (VASP)^34^,^35^ with projector-augmented wave (PAW) potential^36^. The exchange-correlation functional is chosen to be that given by generalized gradient approximation (GGA)^37^. Correlation effects beyond GGA are taken into account through an on-site Hubbard *U* correction in form of GGA +*U*^38^. Since we are not interested in the magnetism of Gd spins in the present study, in the plane-wave calculations for the results reported here, the Gd *f* electrons are considered to be part of the core orbitals. The use of GGA +*U* turn out to be crucial for the correct description of the Mott insulating behavior of GTO. The GGA+*U* implementation in the plane wave code of VASP with PAW potential needed a *U* value of 7 eV applied on Ti *d* states, for the bulk GTO to be insulating. The Hund’s coupling parameter *J*_*H*_ is chosen be 1 eV. We have thus consistently used *U* = 7 eV and *J*_*H*_ = 1 eV on Ti *d* states throughout our calculations. We found a smaller *U* value (≈4 eV) within the linear muffin tin orbital (LMTO) basis calculation to be sufficient to drive bulk GTO insulating, a value more consistent with spectroscopic considerations^39^. The fact that different *U* values are needed in different basis set implementations of DFT has been appreciated in the literature (see, e.g., ref. 40). We found in our LMTO calculations of bulk GTO that taking into account the effect of *U* on both Ti *d* and Gd *f* states, with choice of *U*_*Ti*_ = 4 eV and *U*_*Gd*_ = 9 eV, and *J*_*H*_ = 1 eV, resulted into a ferrimagnetic ground state with antiparallel alignment of the Ti and Gd spins, consistent with experiment.

The in-plane lattice constants of the simulation cells are fixed at the experimental lattice constant of STO with a value of 3.91 Å, while the out-of-plane lattice constant is allowed to relax. The relaxed out-of-plane lattice constants for GTO/STO is found to be ≈2% larger than that of LAO/STO. The consideration of tilt and rotation of metal-oxygen octahedra becomes rather important for GTO with significant orthorhombic distortion. In order to take into account of that, the in-plane dimension of the simulation cell is expanded by 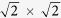. Internal positions of the atoms are allowed to relax until the forces become less than 0.01 eV/Å.

For the calculations carried out on thin film geometry, the thickness of vacuum layer is chosen to be 16 Å. The effect of the artificial electric field in the vacuum due to periodic boundary condition is taken into account through dipole correction, as implemented in VASP. The effect of this artificial electric field is however small, as shown in the detailed calculation by Chen *et al.*^21^ in Appendix B1 of their paper. Considering the dielectric constants of STO and that of LAO and GTO, as shown by Chen *et al.*^21^ the field in STO is found to be only about 0.3% of that in LAO or GTO for a vacuum thickness of 16 Å. Further to establish the convergence of our calculations in terms of vacuum layer thickness, we have carried out additional calculations considering an increased thickness of vacuum layer (30 Å). We found our conclusions concerning the carrier density, minimum thickness of conductivity to remain unchanged, justifying physical soundness of our calculations. The situation becomes different in presence of external electric field which is not considered in the present study.

## Additional Information

**How to cite this article**: Banerjee, H. *et al.* Electronic Structure of Oxide Interfaces: A Comparative Analysis of GdTiO_3_/SrTiO_3_ and LaAlO_3_/SrTiO_3_ Interfaces. *Sci. Rep.* **5**, 18647; doi: 10.1038/srep18647 (2015).

## Supplementary Material

Supplementary Information

## Figures and Tables

**Table 1:** The layer-wise contribution of the conduction electron in GTO/STO and LAO/STO in superlattice and thin film-substrate geometries.

LAO/STO			GTO/STO		
Superlattice		Thin film-substrate	Superlattice		Thin film-substrate
(LAO)_1.5_/(STO)_4.5_	(LAO)_1.5_/(STO)ub	(LAO)_5_/(STO)	(GTO)_1.5_/(STO)ub	(GTO)_1.5_/(STO)ub	(GTO)_1_/(STO)
Layer: Charge	Layer: Charge	Layer: Charge	Layer: Charge	Layer: Charge	Layer: Charge
2 : 0.185 (1^*st*^ IF)	2 : 0.200 (1^*st*^ IF)	6 : 0.062 (IF)	2 : 0.181 (1^*st*^) IF	2 : 0.163 (1^*st*^ )IF	2 : 0.185 (IF)
3 : 0.184	3 : 0.104	7 : 0.050	3 : 0.203	3 : 0.101	3 : 0.108
4 : 0.260	4 : 0.070	8 : 0.006	4 : 0.231	4 : 0.058	4 : 0.034
5 : 0.184	5 : 0.066	9 : 0.007	5 : 0.203	5 : 0.082	5 : 0.045
6 : 0.185 (2^*nd*^ IF)	6 : 0.060	10 : 0.005	6 : 0.181 (2^*nd*^ IF)	6 : 0.096	6 : 0.049
	7 : 0.060	11 : 0.004		7 : 0.096	7 : 0.044
	8 : 0.066	12 : 0.002		8 : 0.082	8 : 0.029
	9 : 0.070	13 : 0.001		9 : 0.058	9 : 0.006
	10 : 0.104	14 : 0.000		10 : 0.101	10 : 0.000
	11 : 0.200 (2^*nd*^ IF)			11 : 0.163 (2^*nd*^ IF)	
Total Charge: 0.999	Total Charge: 1.000	Total Charge: 0.137	Total Charge: 0.999	Total Charge: 1.000	Total Charge: 0.500

In case of superlattices, results for both (LAO or GTO)_1.5_/(STO)_4.5_ and (LAO or GTO)_1.5_/(STO)_9.5_ are shown. For thin film-substrate geometry, we show the results for LAO (5 u.c.) and GTO (1 u.c.), the minimum thicknesses in each case at which the 2DEG is formed.
