# Supplementary material for: Electronic Structure of Oxide Interfaces: A Comparative Analysis of GdTiO3/SrTiO3 and LaAlO3/SrTiO3 Interfaces
Source: Sci Rep. 2015 Dec 22;5:18647. doi: 10.1038/srep18647 (PMC4686895; doi:10.1038/srep18647)
Supplement: Supplementary Information [file srep18647-s1.pdf]

Supplementary Information

Electronic Structure of Oxide Interfaces: A Comparative Analysis of $GdTiO_3/SrTiO_3$ and $LaAlO_3/SrTiO_3$ Interfaces

Hrishit Banerjee[1], Sumilan Banerjee[2], Mohit Randeria[3], and Tanusri Saha-Dasgupta[1]

[1]*Department of Condensed Matter Physics and Material Sciences,*

S.N. Bose National Centre for Basic Sciences, JD Block,

Sector-III, Salt Lake City, Kolkata 700 098, India

[2]*Department of Condensed Matter Physics,*

Weizmann Institute of Science, Israel, 7610001 and

[3]*Department of Physics, Ohio State University, Columbus, OH 43210, United States*

PACS numbers: 73.20.-r,71.20.-b,71.20.Be

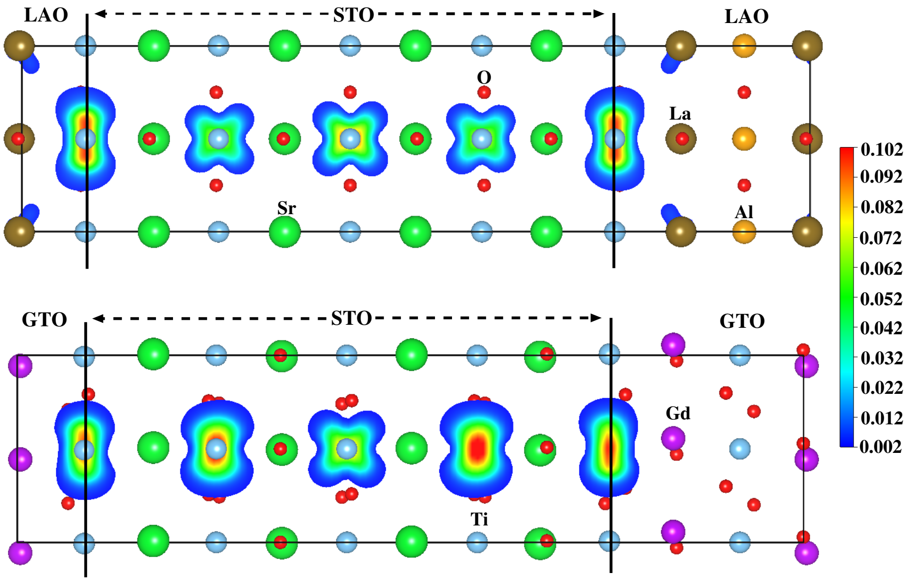

FIG. 1: Conduction electron charge density for $(LAO)_{1.5}/(STO)_{4.5}$ (upper panel) and $(GTO)_{1.5}/(STO)_{4.5}$ (lower panel) in superlattice geometry, integrated over an energy window from 0.1 eV below E_F to E_F. The color of different contours corresponds to the values shown in the scale bar.

In this supplementary, we provide additional information on (a) orbital structure of the conduction electron, in case of LAO/STO and GTO/STO superlattices, as reflected in the charge density plot, and (b) spin-polarization of Ti d states in GTO/STO heterostructures both in superlattice and thin film-substrate geometries.

In particular, in Fig. S1 we show conduction electron charge density for $(LAO)_{1.5}/(STO)_{4.5}$ and $(GTO)_{1.5}/(STO)_{4.5}$ in superlattice geometry, integrated over an energy window from 0.1 eV below E_F to E_F, and in Fig S2 we show spin-polarized DOS projected onto Ti d states for GTO/STO in superlattice and thin film-substrate geometry.

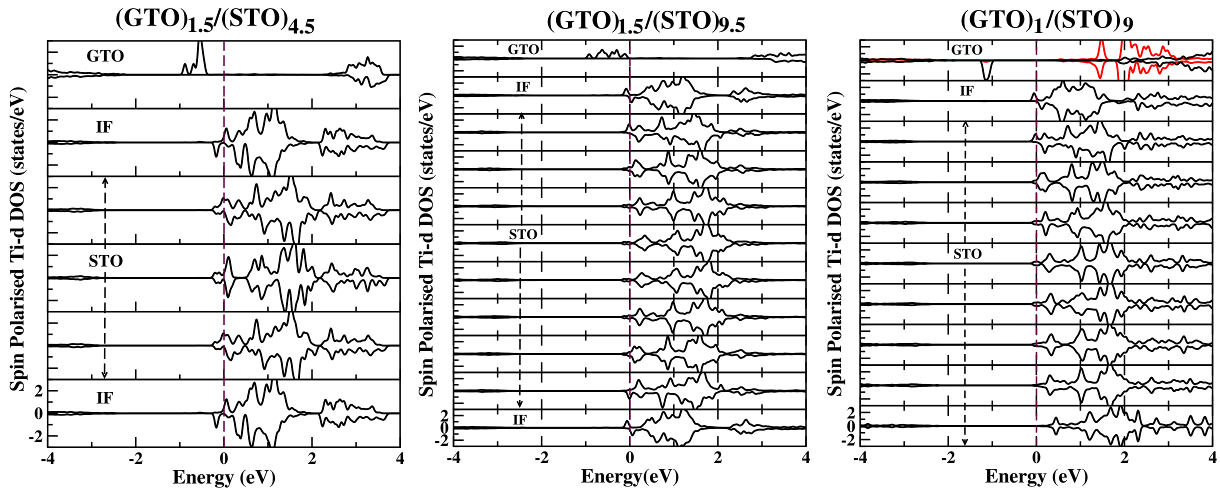

FIG. 2: Spin-polarized DOS projected onto Ti d states for GTO/STO in superlattice (left and middle panels) and thin film-substrate geometry (right panel). For the thin film-substrate geometry, in the topmost TiO_2 layer, which is the surface layer of GTO, the projection to two charge disproportionate Ti d states are shown in black and red lines. The zero of the energy is set at Fermi level.
